# A comprehensive integrated post-GWAS analysis of Type 1 diabetes reveals enhancer-based immune dysregulation

**DOI:** 10.1371/journal.pone.0257265

**Published:** 2021-09-16

**Authors:** Seung-Soo Kim, Adam D. Hudgins, Jiping Yang, Yizhou Zhu, Zhidong Tu, Michael G. Rosenfeld, Teresa P. DiLorenzo, Yousin Suh

**Affiliations:** 1 Department of Obstetrics and Gynecology, Columbia University Irving Medical Center, New York, New York, United States of America; 2 Department of Genetics and Genomic Sciences, Icahn School of Medicine at Mount Sinai, New York, New York, United States of America; 3 Howard Hughes Medical Institute, Department of Medicine, University of California, San Diego, La Jolla, California, United States of America; 4 Department of Microbiology and Immunology, Albert Einstein College of Medicine, Bronx, New York, United States of America; 5 Division of Endocrinology, Department of Medicine, Albert Einstein College of Medicine, Bronx, New York, United States of America; 6 Einstein-Mount Sinai Diabetes Research Center, Albert Einstein College of Medicine, Bronx, New York, United States of America; 7 The Fleischer Institute for Diabetes and Metabolism, Albert Einstein College of Medicine, Bronx, New York, United States of America; 8 Department of Genetics and Development, Columbia University Irving Medical Center, New York, New York, United States of America; Children’s Hospital Boston, UNITED STATES

## Abstract

Type 1 diabetes (T1D) is an organ-specific autoimmune disease, whereby immune cell-mediated killing leads to loss of the insulin-producing β cells in the pancreas. Genome-wide association studies (GWAS) have identified over 200 genetic variants associated with risk for T1D. The majority of the GWAS risk variants reside in the non-coding regions of the genome, suggesting that gene regulatory changes substantially contribute to T1D. However, identification of causal regulatory variants associated with T1D risk and their affected genes is challenging due to incomplete knowledge of non-coding regulatory elements and the cellular states and processes in which they function. Here, we performed a comprehensive integrated post-GWAS analysis of T1D to identify functional regulatory variants in enhancers and their cognate target genes. Starting with 1,817 candidate T1D SNPs defined from the GWAS catalog and LDlink databases, we conducted functional annotation analysis using genomic data from various public databases. These include 1) Roadmap Epigenomics, ENCODE, and RegulomeDB for epigenome data; 2) GTEx for tissue-specific gene expression and expression quantitative trait loci data; and 3) lncRNASNP2 for long non-coding RNA data. Our results indicated a prevalent enhancer-based immune dysregulation in T1D pathogenesis. We identified 26 high-probability causal enhancer SNPs associated with T1D, and 64 predicted target genes. The majority of the target genes play major roles in antigen presentation and immune response and are regulated through complex transcriptional regulatory circuits, including those in HLA (6p21) and non-HLA (16p11.2) loci. These candidate causal enhancer SNPs are supported by strong evidence and warrant functional follow-up studies.

## Introduction

Type 1 diabetes (T1D) is a chronic autoimmune disease with a T cell-mediated loss of functional pancreatic β-cell mass [1, 2]. T1D is divided into three serial stages according to the number of autoantibodies and onset of clinical T1D symptoms: stage 1, presence of two or more autoantibodies with normoglycemia; stage 2, two or more autoantibodies with dysglycemia; and stage 3, significant hyperglycemia with classic T1D symptoms such as polyuria, polydipsia, weight loss, fatigue, diabetic ketoacidosis (DKA), and other complications [3].

Under normal conditions, self-reactive T cells are either eliminated by central tolerance induction in the thymus or are controlled by peripheral tolerance mechanisms, including suppression by regulatory T (T_REG_) cells. Several hypotheses regarding the onset and development of T1D have been suggested. These include the idea that T1D is caused by impaired thymic deletion of autoreactive T cells and/or dysfunctional T_REG_-mediated suppression of autoreactive T cells [4], or that T_REG_-resistant hyperactivated effector T cell populations cause T1D by attacking β-cells in the pancreas [5, 6]. At the onset of T1D in non-obese diabetic mice, innate immune cells such as macrophages and dendritic cells infiltrate the islets of Langerhans [7], where they can acquire autoantigens and subsequently stimulate T cell responses [2].

A well-established T1D genetic risk region is the chromosome 6 human leukocyte antigen (HLA) locus [8, 9], which contributes about 50% of the total genetic risk of T1D [10]. Several studies have established associations between HLA haplotypes and the incidence of β-cell autoantibodies [11, 12]. To better understand how genetic variation might affect the risk and progression of T1D, including the contributions by non-HLA genetic factors [10, 13], genome-wide association studies (GWAS) have been conducted by many groups, including the Type 1 Diabetes Genetics Consortium (T1DGC) [14], and have discovered > 60 T1D susceptibility regions [8]. The data from these and other studies have been recently collected and made available at the T1D Knowledge Portal (https://t1d.hugeamp.org), a creation of the Accelerating Medicines Partnership- Type 2 Diabetes consortium. As observed in all GWAS analysis [15], 90% of GWAS-identified T1D variants are located in non-coding regions, implying that gene regulatory changes underlie genetic risk of T1D. Non-coding variants in gene regulatory elements such as enhancers, promoters, long non-coding RNAs (lncRNAs), and 5′ and 3′ untranslated regions have the potential to dysregulate gene expression through *cis* or *trans* mechanisms [16–18]. However, identification of causal regulatory variants and affected target genes using the sequence information alone is challenging.

Many recent studies show that non-coding GWAS variants are significantly enriched in cell type-specific transcriptional enhancer regions [19–24]. Enhancers have emerged as major points of integration of intra- and extra-cellular signals associated with development, homeostasis, and disease, resulting in context-specific transcriptional outputs [25, 26]. The human genome is estimated to encode ~1 million enhancer elements, with distinct sets of approximately 30,000–40,000 enhancers being active in a particular cell type [27]. Cell type-specific enhancer activation is driven by combinatorial actions of lineage-determining and signal-dependent transcription factors (TFs) [26, 28]. Genetic variation affecting enhancer selection and function is considered to be a major determinant of differences in cell type-specific gene expression and disease risk between individuals [29]. Indeed, multiple studies have shown that T1D-associated risk variants are enriched in immune cell-specific enhancer regions [24, 30, 31], and a recent study reported dysregulation of enhancer function in type 1 helper T and T_REG_ cells of T1D patients, with decreased target gene expression levels [32].

Here, to identify causal enhancer variants associated with T1D, we conducted an integrated analysis of GWAS-identified T1D variants by utilizing a comprehensive set of public databases, including epigenomic datasets (e.g., Roadmap Epigenomics project and ENCODE), expression quantitative trait loci (eQTL) data from the Genotype-Tissue Expression project (GTEx), and a non-coding RNA (ncRNA) database (lncRNASNP2). Our results indicate that T1D-associated non-coding variants may contribute to T1D risk by mediating enhancer-based immune dysregulation. We highlight 26 high-probability causal enhancer variants associated with T1D risk and demonstrate complex transcriptional regulatory circuits at both HLA and non-HLA loci mediated through these variants.

## Materials and methods

### Identification of candidate T1D SNPs

From the GWAS Catalog database [33], a curated T1D-associated single nucleotide polymorphism (SNP) set was retrieved and filtered by p-value < 5E-8 (n = 129, download date: 11/5/2018). SNPs in high linkage disequilibrium (LD) with T1D GWAS SNPs were collected using the web-based application LDlink [34] and filtered by European sub-population (CEU, TSI, FIN, GBR, IBS) LD criteria (r^2^ > 0.6 and D’ = 1, n = 1,686). The combined 1,817 candidate T1D SNPs, with coordinates mapped to genome build hg19, were used for the following analysis.

### Enhancer SNP prioritization and annotation of long non-coding RNA (lncRNA) co-localization

The Roadmap Epigenomics project (http://www.roadmapepigenomics.org/) provides genome-wide epigenomic annotations using the ChromHMM algorithm [35]. The ChromHMM epigenome annotation data used for variant annotation was filtered by enhancer tags (e.g., 13_EnhA1, 14_EnhA2, 15_EnhAF, 16_EnhW1, 17_EnhW2, and 18_EnhAc). To identify transcription factor binding site (TFBS) regions, ENCODE ChIP-seq data was downloaded from the UCSC Genome Browser (http://genome.ucsc.edu/; file name: wgEncodeRegTfbsClusteredV3.bed.gz).

Using bedtools (https://bedtools.readthedocs.io/en/latest/), Roadmap enhancer regions were merged to avoid duplicated counts from overlapped enhancer regions, and the distance from a candidate SNP to the closest enhancer was calculated by using the ‘merge’ and ‘closest’ functions in bed tools. Next, candidate SNPs were annotated as being located within an enhancer region by having a distance of 0. The same procedure was repeated for the ENCODE TFBS regions to find TFBS-occupied SNPs.

The public database RegulomeDB [36] provides category scores for SNPs by using curated evidence. The T1D candidate SNPs were stringently filtered by their scores (i.e., ≥ 2b), including only SNPs with functional motif evidence (e.g., TF binding, any motif, DNase Footprint, and DNase peak, see details at http://www.regulomedb.org/index).

Using lncRNASNP2 (http://bioinfo.life.hust.edu.cn/lncRNASNP), which provides comprehensive information on SNPs located in long non-coding RNA (lncRNA) coding regions [37], the T1D candidate SNPs located in lncRNA coding regions were selected. The full R scripts and processed files for this analysis were uploaded to the GitHub repository (https://github.com/kisudsoe/SNP_prioritization_T1D).

### Identification of eQTLs and nearest genes

Full data of the Genotype-Tissue Expression (GTEx) project (Version 7, 09/05/2017) [16] was downloaded (https://gtexportal.org/home/). GTEx V7 includes 11,688 samples of 53 tissues from 714 donors. The eQTL-gene association data was filtered by nominal p-value < 5E-8. The nearest genes of the eQTLs were searched from the total gene regions registered in Ensembl DB (Version: GRCh37.p13) by using the ‘closest’ function in bedtools.

### Gene ontology (GO) analysis, functional categorization and tissue-specific gene expression analysis

From the list of 159 eQTL-associated genes, significantly enriched terms from the three GO categories (biological process (BP), cellular component (CC), and molecular function (MF)), as well as KEGG pathways, were identified using the web-based utility DAVID Bioinformatic Resource 6.8 (https://david.ncifcrf.gov/home.jsp), with a false discovery rate (FDR) threshold of 0.05 [38]. By using significant GO terms and KEGG pathways, as well as gene function descriptions in Genecards (https://www.genecards.org), genes were categorized into 20 functional groups. To identify tissue-specific gene expression patterns, the 159 genes were clustered by their tissue median TPM values from GTEx RNA-seq data, using the ‘hclust’ function in R (cluster cut by distance = 7.6).

### Tissue/Cell type-specific enrichment and physical interactions of candidate T1D SNPs

SNPsea [39] was used to calculate the tissue/cell type-specific enrichment of T1D risk loci gene expression. SNPsea uses linkage disequilibrium (r^2^ > 0.5) data from the EUR population of the 1000 Genomes Project [40] to identify the genes implicated by the input SNPs. In this study, the 1,817 candidate T1D SNPs were used as input data, and the default tissue/cell type-specific gene expression datasets were used (i.e. FANTOM5 [41] and GeneAtlas2004 [42]). The CoDeS3D algorithm [43] was used to identify spatial SNP-gene interactions (FDR < 0.05) using the list of 1,817 candidate T1D SNPs, Hi-C data from human cell lines including IMR90, HMEC, NHEK, KBM7, and HUVEC [44] and GTEx eQTL data [16].

## Results

### An integrated functional annotation analysis identifies candidate T1D enhancer SNPs and risk genes

**Fig 1:**
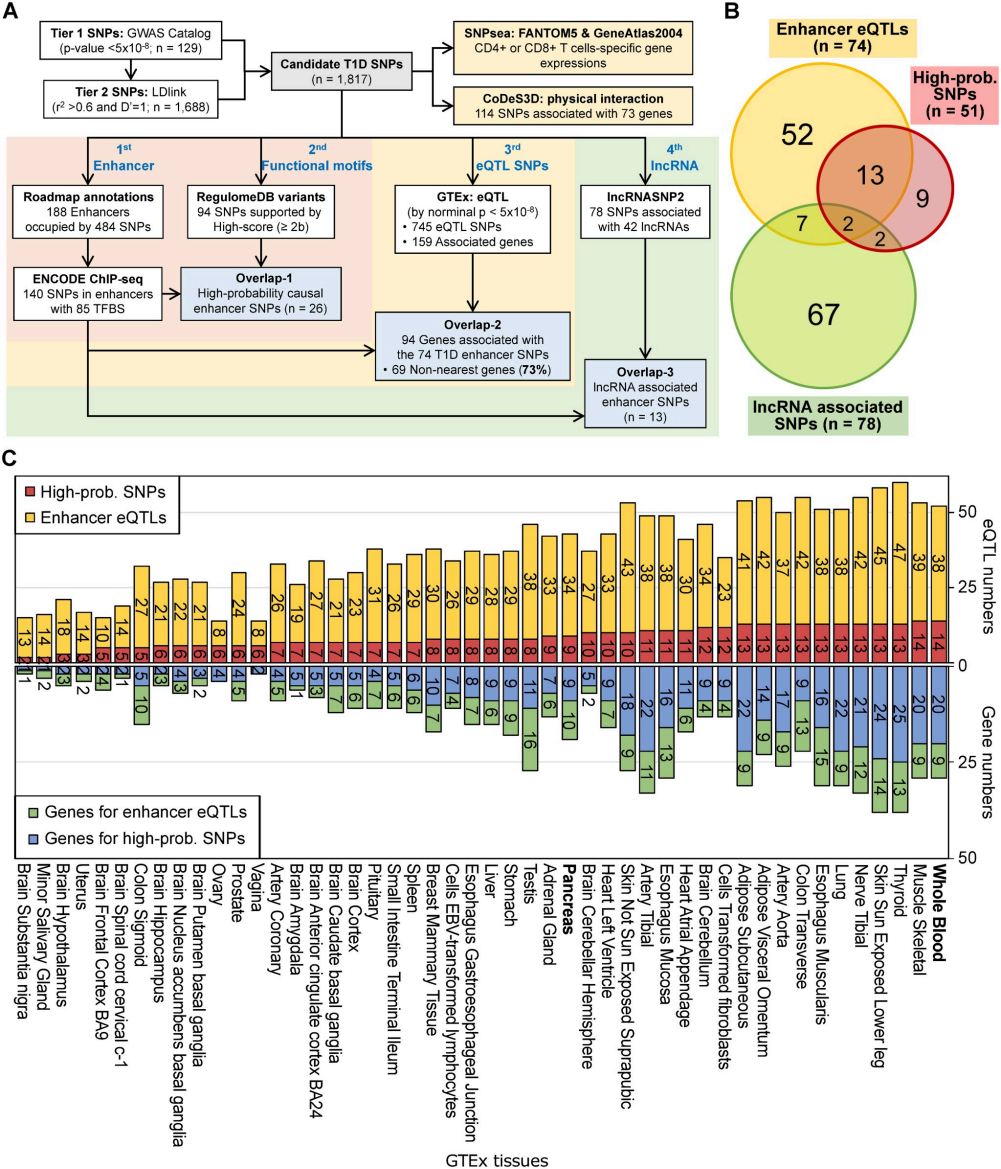
Overview of T1D SNP integrative annotation analyses. **A**. Candidate T1D SNPs (n = 1, 817) comprised both GWAS-identified SNPs (Tier 1, n = 129, p <5E-8) and SNPs in high LD (Tier 2, n = 1,688, r^2^ > 0.6 and D′ = 1). To prioritize high-confidence candidate T1D SNPs, four independent analyses, involving five public DBs, were conducted: 1) Integration of Roadmap enhancer and ENCODE TFBS annotations to identify functional enhancer SNPs; 2) Querying RegulomeDB to find functional evidence of causal T1D SNPs; 3) Mining GTEx data to identify eQTL-gene associations for T1D SNPs; 4) Utilizing lncRNASNP2 data to identify T1D SNPs located in lncRNA regions. Independently, the SNPsea algorithm was applied to the candidate T1D SNPs to identify tissue-specific enrichment of T1D risk loci gene expression, and the CoDeS3D algorithm was applied to find evidence of potential physical interactions between candidate T1D SNPs and risk locus genes. **B**. Venn diagram showing the overlap between prioritized T1D SNPs, GTEx enhancer eQTLs, and lncRNASNP2 lncRNA data. Among the 26 high-probability causal enhancer T1D SNPs, we found 15 eQTLs associated with 64 downstream genes (S3 Table), and 4 SNPs located in 5 lncRNAs (S4 Table). The highest priority SNPs, rs3129716 and rs886424, are both GTEx eQTLs and are located in lncRNA regions. **C**. Tissue distribution of GTEx eQTLs and their corresponding genes. Of the 48 tissues, whole blood and skeletal muscle have the largest numbers (14 SNPs) of eQTLs, with 20 genes implicated by the high-probability SNPs. In pancreas, there are 9 eQTLs among the high-probability SNPs, with 9 corresponding genes. The numbers of enhancer eQTLs and high-probability SNPs are colored as yellow and red, respectively. The numbers of genes paired with enhancer eQTLs and high-probability SNPs are colored as green and blue, respectively. Whole blood and pancreas are marked in bold.

Since genetic variation in enhancer elements has been found to be major determinants of the genetic risk for many complex diseases [29], we hypothesized that T1D-associated non-coding variants located in enhancers are strong candidates for the causal variants underlying T1D risk. To identify these candidate functional enhancer variants, we conducted an integrative analysis of SNPs detected by T1D GWAS as well as SNPs in linkage disequilibrium (LD) with T1D-associated GWAS lead SNPs. First, we retrieved T1D-associated GWAS lead SNPs from the GWAS catalog (p-value < 5E-8), here referred to as Tier 1 SNPs (n = 129). We then expanded this list to include SNPs in LD with the lead SNPs, referred to as Tier 2 SNPs (n = 1,688), based on stringent LD criteria (r^2^ > 0.6 and D’ = 1) by using LDlink and European population genetic data from the 1000 Genomes Project. As a result, we compiled 1,939 pairs of lead SNPs-LD linked SNPs (S1 Table), and 1,817 candidate T1D SNPs (Fig 1).

As a first step in our integrative analysis, we annotated our candidate T1D SNPs using the Roadmap Epigenomics project datasets and found that 188 enhancer regions are occupied by 484 candidate T1D SNPs (Fig 1A). To increase the functional relevance of our analysis, we utilized ENCODE ChIP-Seq data to further examine which of the enhancers co-localized with transcription factor binding sites (TFBS). We found a total of 85 candidate T1D SNP-occupied enhancers with effective TF binding, and 140 candidate T1D SNPs residing in these enhancers (Fig 1A and S2 Table). In addition, we also utilized the RegulomeDB database to assign functional scores to the candidate T1D SNPs, resulting in 94 SNPs with a score ≥ 2b (with evidence of TF-binding, any TF motif, DNase Footprint, and DNase peak signal). Among the 94 SNPs, 26 SNPs are located within TFBS-containing enhancers, and were prioritized as “high-probability” causal enhancer variants (Fig 1B, Table 1 and S2 Table).

**Table 1:** High-probability causal enhancer SNPs, eQTLs, and lncRNAs.

Rsid	DB source	Chr	Position	High-probability causal enhancer SNPs^1^		ENCODE Canonical TF-binding Motifs
				GTEx eQTLs Gene#^2^	lncRNASNP2 lncRNA#^3^	
**rs886424**	**GWAS**	**chr6**	**30782002**	**11**	**2**	**-**
**rs3129716**	**LDlink**	**chr6**	**32657436**	**14**	**1**	**-**
**rs1264361**	**LDlink**	**chr6**	**30777498**	**11**	-	-
**rs9268606**	**LDlink**	**chr6**	**32400070**	**7**	-	-
rs1049053	LDlink	chr6	32634405	-	-	-
rs9274626	LDlink	chr6	32636040	-	-	-
rs9388486	LDlink	chr6	126661154	-	1	-
rs3024493	LDlink	chr1	206943968	-	-	-
rs3024505	GWAS	chr1	206939904	-	-	-
rs478222	GWAS	chr2	25301755	3	-	-
rs11715915	LDlink	chr3	49455330	9	-	-
rs6997	LDlink	chr3	49453834	8	-	-
rs9814873	LDlink	chr3	49454112	9	-	-
rs7725052	GWAS	chr5	40487270	-	-	-
rs7731626	GWAS	chr5	55444683	-	-	-
rs68037604	LDlink	chr11	2212487	-	1	-
rs10876870	LDlink	chr12	56478002	3	-	-
rs4759229	LDlink	chr12	56474480	3	-	-
**rs4788084**	**GWAS**	**chr16**	**28539848**	**17**	**-**	**EBF1**
**rs62031562**	**LDlink**	**chr16**	**28609329**	**18**	**-**	**-**
**rs743590**	**LDlink**	**chr16**	**28608230**	**18**	**-**	**-**
**rs762633**	**LDlink**	**chr16**	**28608341**	**18**	**-**	**-**
rs12919083	LDlink	chr16	11188930	-	-	-
rs7203793	LDlink	chr16	11182134	-	-	-
rs3746923	LDlink	chr21	43826344	-	-	-
rs229544	LDlink	chr22	37593080	1	-	MAX, USF1

**Fig 2:**
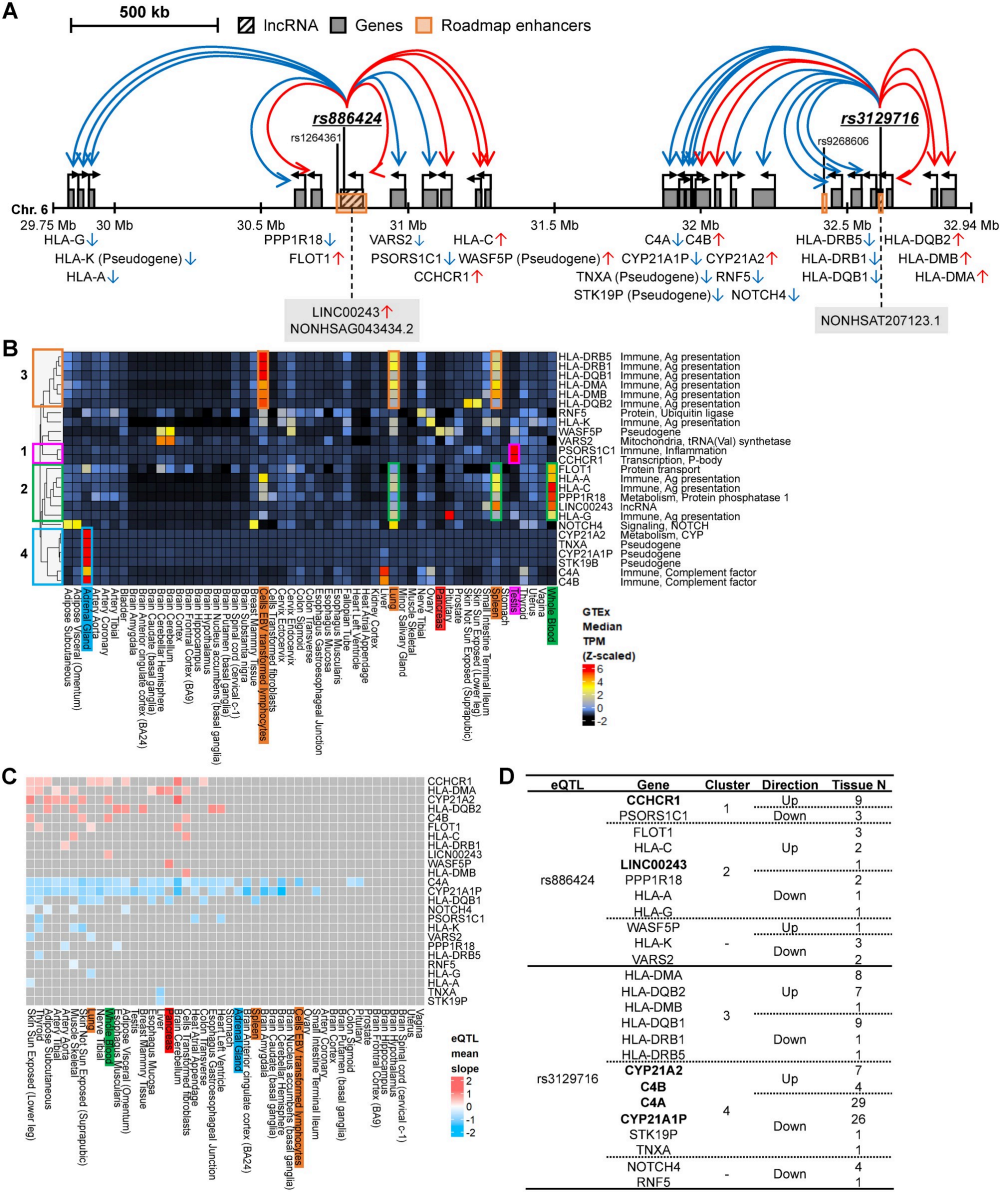
Complex transcriptional regulatory circuits in the HLA locus (6p21). **A**. Regulatory circuits of the high-probability causal enhancer T1D SNPs, rs886424 and rs3129716, in the chromosome 6 HLA locus, including 25 associated genes. Red colored arrows indicate that the SNP is associated with increased expression of the indicated gene and blue colored arrows indicate an association with decreased expression of the indicated gene, according to GTEx eQTL data. The two eQTLs are located at lncRNA regions NONHSAG043434.2 (HGNC Symbol: *LINC00243*) and NONHSAT207123.1, respectively. **B**. Relative median gene expression levels of the 25 genes in the HLA locus from GTEx RNA-seq data. Based on their tissue-specific expression patterns, four representative gene groups were discovered by hierarchical clustering. The genes highly expressed in each organ are marked with colored boxes corresponding to the clustered groups. **C**. A heatmap for T1D-associated eQTL effect size, using the slope values of the eQTL-gene pairs from the GTEx eQTL analysis. **D**. A summary table of the two eQTLs rs886424 and rs3129716, including their 25 associated genes (“Gene” column), hierarchical clustering results of the tissue-specific gene expressions (“Cluster” column), directional effects of eQTLs on the genes (“Direction” column), and tissue numbers affected by eQTLs (“Tissue N” column). The genes with whole blood eQTLs are marked as bold font.

**Fig 3:**
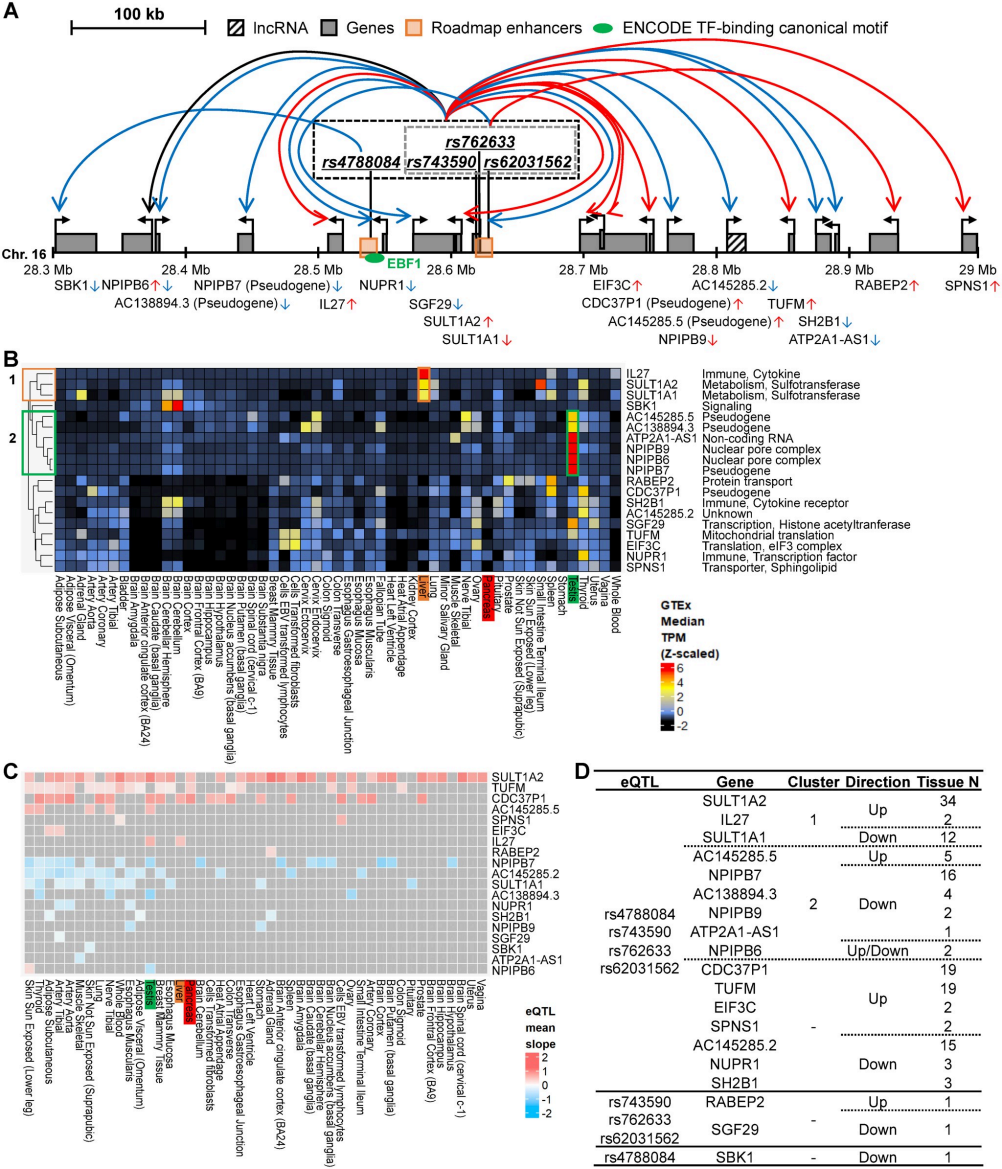
Complex transcriptional regulatory circuits in the 16p11.2 locus. **A**. This diagram shares the same rules for color, shape, and arrows as Fig 2. Regulatory circuits of the four T1D-associated eQTLs (rs4788084, rs762633, rs743590, and rs62031562) and their 19 associated genes. The four eQTLs share a common direction of association with 16 of the 19 genes. Two of the genes, *SGF29* and *RABEP2*, are associated with three of the eQTLs, rs762633, rs743590, and rs62031562. The expression of *SBK1* is associated only with rs4788084. The enhancer region harboring rs4788084 contains a canonical binding motif for the TF EBF1. **B**. Relative gene expression levels of the 19 genes are displayed as a heatmap. By hierarchical clustering, two representative groups were found with high tissue-specific expression patterns. The genes highly expressed in each organ are marked with colored boxes corresponding to the clustered groups. **C**. A heatmap for T1D-associated eQTL effect size, using the slope values of the eQTL-gene pairs from the GTEx eQTL analysis. **D**. A summary table of the four eQTLs rs4788084, rs743590, rs762633 and rs62031562, including their 19 associated genes (“Gene” column), hierarchical clustering results of the tissue-specific gene expressions (“Cluster” column), directional effects of eQTLs on the genes (“Direction” column), and tissue numbers affected by eQTLs (“Tissue N” column).

Prioritized T1D SNPs are enriched in chromosome 6 and 16 which are marked with gray shading. Rs886424, rs3129716, rs1264361, and rs9268606 in the HLA region (chromosome 6p21.33, see Fig 2A) and rs4788084, rs62031562, rs743590, and rs762633 in chromosome 16p11.2 locus (see Fig 3A) are marked as bold font.

^1^Integration of the Roadmap, ENCODE ChIP-seq, and RegulomeDB.

^2^64 genes are associated with 15 T1D eQTLs. See full list of gene-eQTL pairs in S3 Table.

^3^4 SNPs are located within 5 lncRNA regions. See full list of lncRNA-SNP pairs in S6 Table.

We next investigated whether any of the candidate T1D SNPs are eQTLs by querying data from the Genotype-Tissue Expression (GTEx) project Portal. A total of 745 candidate T1D SNPs have significant eQTL associations with 159 genes (p < 5E-8), among which 94 genes are associated with 74 SNPs residing in enhancers with effective TF binding, indicating that over half of the genes (94/159 genes, 59%) are associated with eQTLs residing in enhancers (Fig 1A and 1B, and S3 Table). Out of the 48 GTEx tissues, 14 and 9 tissue-specific eQTLs were found from the high-probability SNPs in whole blood (Rank 1) and pancreas (Rank 20), respectively (Fig 1C). In standard GWAS analysis, the genes in closest proximity to the disease-associated SNPs are assigned as the causal genes. However, our results indicated that the majority of the 94 genes found through the eQTL analysis (69/94 genes, 73%) are not the nearest genes to the candidate T1D SNPs, suggesting that the conventional “nearest gene” method of assigning target genes to enhancers can be inaccurate and misleading, as previously reported [45, 46].

By utilizing data from chromosome conformation capture studies, further information regarding the real target genes of enhancer SNPs can be revealed. Using publicly available Hi-C data (see details in Materials and methods section), we used the CoDeS3D algorithm [43] to search for potential physical interactions between SNP-gene pairs in the 1,817 candidate T1D SNPs. We found that 73 genes are spatially associated with 114 T1D SNPs (S4 Table), among which 24 genes (6 HLA and 18 non-HLA genes) overlap with the 159 genes found through the eQTL analysis. 54% (13/24 genes) of the CoDeS3D-identified genes are involved in the immune response (see “CoDeS3D” column in S5 Table).

Finally, non-coding SNPs can occur in the open reading frames of lncRNAs, with potential impacts not only on lncRNA expression but also lncRNA structure and function [47, 48]. To identify functional T1D variants potentially affecting lncRNAs, we queried the lncRNASNP2 database and found that 78 candidate T1D SNPs reside in 42 lncRNAs (Fig 1A and 1B, and S6 Table). Out of these 78 SNPs, 13 are enhancer SNPs, among which rs3129716 and rs886424 are two of the 26 high-probability causal enhancer SNPs (Table 1). These two candidate T1D SNPs are located in the chromosome 6 HLA region and are eQTLs for 25 nearby genes; rs3129716 is an eQTL for 14 genes and rs886424 is an eQTL for 11 genes (Fig 2A).

### Candidate T1D risk genes are related to immune response and exhibit tissue-specific expression

To gain insights into the biological functions of the 159 candidate T1D risk genes identified through the eQTL analysis, we performed a gene ontology (GO) enrichment analysis using the web-based DAVID utilities (https://david.ncifcrf.gov/home.jsp). We found that the most significant GO terms are related to immune response: regulation of immune response (GO:0050776, FDR = 6.9E-10) in the Biological Process (BP) category; Major Histocompatibility Complex (MHC) protein complex (GO:0042611, FDR = 1.4E-12) in the Cellular Component (CC) category; and MHC class II receptor activity (GO:0032395, FDR = 2.5E-7) in the Molecular Function (MF) category. Other significant GO terms are also in immune response-related functions, such as antigen processing and presentation, interferon-gamma-mediated signaling pathway, and T cell activation (full list of the GO terms in S7 Table). Consistently, the significantly enriched KEGG (Kyoto Encyclopedia of Genes and Genomes) pathways include antigen processing and presentation (hsa04612, FDR = 8.9E-9), and cell adhesion molecules (hsa04514, FDR = 1.6E-8) (full list of the KEGG pathways in S7 Table).

Next, the 159 candidate T1D risk genes were subdivided into different functional categories (see details in Materials and methods), with the largest category being immune response (57 genes, 36%), including 23 genes involved in antigen presentation, 18 genes in immune signaling, 9 genes in adaptive immunity, 5 genes in innate immunity, and 2 genes related to other immune-related functions (Table 2). Other functional categories included metabolic enzymes (13 genes), transcription (9 genes), signaling (8 genes), protein metabolism (7 genes), RNA metabolism (6 genes), mitochondria (5 genes), and others (26 genes) (S6 Table). The 26 high-probability causal enhancer SNPs that we identified through integrated functional annotation analysis (Fig 1) are associated with 64 genes by eQTL analysis. Of these 64 genes, 26 (41%) are immune response genes, of which 11 (48% of 26 genes) have eQTL associations in whole blood (see “High_prob_SNP” and “Tissue” columns in S6 Table and Table 2). In addition, there are 10 pseudogenes and 18 genes with unknown function.

**Table 2:** Genes involved in immune response represent the largest functional category of eQTL-associated genes.

Ensembl ID	Symbol	Name	Note	High-probability SNPs	Hclust group*
**Antigen presentation**					
ENSG00000204520.8	*MICA*	MHC class I polypeptide-related sequence A	HLA locus	-	3
ENSG00000204525.10	*HLA-C*	Major histocompatibility complex, class I, C	HLA locus	**rs886424**, **rs1264361**	**7**
ENSG00000206503.7	*HLA-A*	Major histocompatibility complex, class I, A	HLA locus	**rs886424**, **rs1264361**	**7**
ENSG00000204632.7	*HLA-G*	Major histocompatibility complex, class I, G	HLA locus	**rs886424**, **rs1264361**	**7**
ENSG00000204516.5	*MICB*	MHC class I polypeptide-related sequence B	HLA locus	-	**8**
ENSG00000196126.6	*HLA-DRB1*	Major histocompatibility complex, class II, DR beta 1	HLA locus	**rs3129716**, **rs9268606**	**8**
ENSG00000237541.3	*HLA-DQA2*	Major histocompatibility complex, class II, DQ alpha 2	HLA locus	**rs9268606**	**8**
ENSG00000232629.4	*HLA-DQB2*	Major histocompatibility complex, class II, DQ beta 2	HLA locus	**rs3129716**, **rs9268606**	**8**
ENSG00000204257.10	*HLA-DMA*	Major histocompatibility complex, class II, DM alpha	HLA locus	**rs3129716**	**8**
ENSG00000242574.4	*HLA-DMB*	Major histocompatibility complex, class II, DM beta	HLA locus	**rs3129716**	**8**
ENSG00000196735.7	*HLA-DQA1*	Major histocompatibility complex, class II, DQ alpha 1	HLA locus	**rs9268606**	**8**
ENSG00000179344.12	*HLA-DQB1*	Major histocompatibility complex, class II, DQ beta 1	HLA locus	**rs3129716**, **rs9268606**	**8**
ENSG00000198502.5	*HLA-DRB5*	Major histocompatibility complex, class II, DR beta 5	HLA locus	**rs3129716**	**8**
ENSG00000176920.10	*FUT2*	Fucosyltransferase 2	H antigen	-	21
ENSG00000223534.1	*HLA-DQB1-AS1*	HLA-DQB1 antisense RNA 1	Non-coding RNA	-	**8**
ENSG00000176998.3	*HCG4*	HLA complex group 4	Non-coding RNA	-	15
ENSG00000206337.6	*HCP5*	HLA complex P5	Non-coding RNA	-	**8**
ENSG00000103811.11	*CTSH*	Cathepsin H	Proteinase	-	**8**
ENSG00000204622.6	*HLA-J*	Major histocompatibility complex, class I, J (pseudogene)	Pseudogene	-	**7**
ENSG00000230795.2	*HLA-K*	Major histocompatibility complex, class I, K (pseudogene)	Pseudogene	**rs886424**, **rs1264361**	**7**
ENSG00000229391.3	*HLA-DRB6*	Major histocompatibility complex, class II, DR beta 6 (pseudogene)	Pseudogene	**rs9268606**	**8**
ENSG00000196301.3	*HLA-DRB9*	Major histocompatibility complex, class II, DR beta 9 (pseudogene)	Pseudogene	**rs9268606**	**8**
ENSG00000237669.1	*HCG4P3*	HLA complex group 4 pseudogene	Pseudogene	-	11
**Signaling for immune response**					
ENSG00000173531.11	*MST1*	Macrophage stimulating 1	-	rs11715915, rs6997, rs9814873	10
ENSG00000133466.9	*C1QTNF6*	C1q and TNF related 6	B-cell receptor	rs229544	3
ENSG00000178188.10	*SH2B1*	SH2B adaptor protein 1	Cytokine receptor	**rs4788084**, **rs62031562**, **rs743590**, **rs762633**	1
ENSG00000105397.9	*TYK2*	Tyrosine kinase 2	Cytokine	-	**7**
ENSG00000197272.2	*IL27*	Interleukin 27	Cytokine	**rs4788084**, **rs62031562**, **rs743590**, **rs762633**	10
ENSG00000204616.6	*TRIM31*	Tripartite motif containing 31	Cytokine	-	20
ENSG00000160856.16	*FCRL3*	Fc receptor like 3	Fc receptor-like	-	**7**
ENSG00000240053.8	*LY6G5B*	Lymphocyte antigen 6 family member G5B	Glycophosphatidylinositol	-	1
ENSG00000204421.2	*LY6G6C*	Lymphocyte antigen 6 family member G6C	Glycophosphatidylinositol	-	16
ENSG00000156711.12	*MAPK13*	Mitogen-activated protein kinase 13	Inflammation	-	16
ENSG00000111540.11	*RAB5B*	RAB5B, member RAS oncogene family	SMAD	rs10876870, rs4759229	3
ENSG00000166949.11	*SMAD3*	SMAD family member 3	SMAD	-	3
ENSG00000005020.8	*SKAP2*	Src kinase associated phosphoprotein 2	Src	-	2
ENSG00000184293.3	*CLECL1*	C-type lectin like 1	T cell costimulator	-	**8**
ENSG00000119919.9	*NKX2*-3	NK2 homeobox 3	T cell differentiation, TF	-	12
ENSG00000213658.6	*LAT*	Linker for activation of T cells	T cell, TCR	-	17
ENSG00000171862.5	*PTEN*	Phosphatase and tensin homolog	T cell, TCR	-	3
ENSG00000105287.8	*PRKD2*	Protein kinase D2	T cell, TCR	-	**7**
**Adaptive immunity**					
ENSG00000110852.4	*CLEC2B*	C-type lectin domain family 2 member B	-	-	3
ENSG00000182179.6	*UBA7*	Ubiquitin like modifier activating enzyme 7	-	rs11715915, rs6997, rs9814873	**7**
ENSG00000163599.10	*CTLA4*	Cytotoxic T-lymphocyte associated protein 4	-	-	**7**
ENSG00000150637.4	*CD226*	CD226 molecule	-	-	**8**
ENSG00000136153.15	*LMO7*	LIM domain 7	-	-	13
ENSG00000172575.7	*RASGRP1*	RAS guanyl releasing protein 1	-	-	14
ENSG00000164068.11	*RNF123*	Ring finger protein 123	-	rs11715915, rs6997, rs9814873	19
ENSG00000224389.4	*C4B*	Complement C4B (Chido blood group)	Complement factor	**rs3129716**	27
ENSG00000244731.3	*C4A*	Complement C4A (Rodgers blood group)	Complement factor	**rs3129716**	27
**Innate immunity**					
ENSG00000187796.9	*CARD9*	Caspase recruitment domain family member 9	-	-	**7**
ENSG00000164062.8	*APEH*	Acylaminoacyl-peptide hydrolase	Acylpeptide hydrolase	rs11715915, rs6997, rs9814873	18
ENSG00000167914.6	*GSDMA*	Gasdermin A	Bactericidal activity	-	16
ENSG00000172057.5	*ORMDL3*	ORMDL sphingolipid biosynthesis regulator 3	Protein binding	-	10
ENSG00000204540.6	*PSORS1C1*	Psoriasis susceptibility 1 candidate 1	Inflammation, Psoriasis	**rs886424**, **rs1264361**	11
**Other immune-related**					
ENSG00000185010.9	*F8*	Coagulation factor VIII	Blood coagulation	-	9
ENSG00000176046.7	*NUPR1*	Nuclear protein 1, transcriptional regulator	Transcription factor	**rs4788084**, **rs62031562**, **rs743590**, **rs762633**	17

See S7 Table for full information of the 159 eQTL genes. Genes in gray shading indicate members of Hclust groups 7 and 8, which are enriched for genes involved in antigen presentation (see S1 Fig). The variants rs886424, rs3129716, rs1264361, and rs9268606, which reside in enhancer regions in the HLA locus (chromosome 6p21.33, see Fig 2A), and rs4788084, rs62031562, rs743590, and rs762633, which reside in enhancer regions in the chromosome 16p11.2 locus (see Fig 3A), are marked as bold font.

*Hclust group: The result of hierarchical clustering by gene expression patterns (see S1 Fig).

To further refine the immune system signature of the 159 candidate T1D risk genes, we next sought to identify tissue-specific gene expression patterns. After performing hierarchical clustering of GTEx RNA-seq median gene expression values from 53 tissues, we found that the 159 candidate T1D risk genes clustered into 28 tissue-specific groups (S1 Fig). Among the various tissue-specific patterns that were identified, we observed that genes from the HLA locus mostly clustered in groups 7 and 8 (18/22 genes, 82%), and showed high levels of expression in Epstein-Barr Virus (EBV)-transformed lymphocyte cells, lung, and spleen. In group 11, 18 genes involved in various functions (e.g. metabolism, structure, nucleus, transcription, immune, and transporter) showed a testis-specific pattern of high expression. We also looked for tissue-specific gene expression signatures by applying the SNPsea algorithm to the 1,817 candidate T1D SNPs and found that genes in T1D-associated loci show significant enrichment of expression in CD4^+^ T cells and CD8^+^ T cells (FANTOM5, *P* < 0.0001; GeneAtlas2004, *P* < 0.0005; S2 Fig and S8 Table).

### Candidate causal T1D enhancer SNPs are associated with complex transcriptional regulatory circuits in HLA and non-HLA T1D risk loci

Enhancers are capable of activating transcription of their target genes at great distances, independent of their location, and a single enhancer can regulate multiple target genes [49]. While they can be highly tissue- and cell type-specific, many enhancers are shared among diverse cell types. And yet, the phenotypic effects of the disease- and trait-associated variants that these shared enhancers harbor might only be mediated through specific, disease-relevant cell types [21, 27, 30]. Thus, we examined the transcriptional networks that are affected by the 26 prioritized high-probability causal enhancer SNPs in greater detail. We found that 15 of these variants are GTEx eQTLs (Table 1), among which >50% (8/15) are located in two loci, 6p21.33 and 16p11.2: 4 eQTLs (rs886424, rs3129716, rs1264361, and rs9268606) are located in the HLA locus (6p21.33), a major T1D genetic risk region, while 4 eQTLs (rs4788084, rs62031562, rs743590, rs762633) are located in 16p11.2.

We found that 2 of the 4 eQTLs in the HLA locus (6p21.33), rs886424 and rs3129716, occur in both enhancer regions and lncRNAs, and are associated with the expression of 11 and 14 genes, respectively (Fig 2A). The majority of these genes (10/25 genes, 40%) are HLA genes involved in antigen presentation, including 6 MHC class II genes (Major Histocompatibility Complex, Class II, DR Beta 1 (*HLA-DRB1*), Major Histocompatibility Complex, Class II, DR Beta 5 (*HLA-DRB5*), Major Histocompatibility Complex, Class II, DQ Beta 1 (*HLA-DQB1*), Major Histocompatibility Complex, Class II, DQ Beta 2 (*HLA-DQB2*), Major Histocompatibility Complex, Class II, DM Alpha (*HLA-DMA*), and Major Histocompatibility Complex, Class II, DM Beta (*HLA-DMB*)), 3 MHC class I genes (Major histocompatibility complex, class I, G (*HLA-G*), Major histocompatibility complex, class I, A (*HLA-A*), and Major histocompatibility complex, class I, C (*HLA-C*)), and a pseudogene (Major histocompatibility complex, class I, K (*HLA-K*)) (Fig 2B). The other 15 genes fall into diverse functional categories, including adaptive immunity (complement *C4A* and *C4B*), inflammation (Psoriasis susceptibility 1 candidate 1 (*PSORS1C1*)), metabolic processes (Protein phosphatase 1 regulatory subunit 18 (*PPP1R18*) and Cytochrome P450 family 21 subfamily A member 2 (*CYP21A2*)), mitochondrial tRNA synthesis (Valyl-tRNA synthetase 2, mitochondrial (*VARS2*)), protein processing (Ring finger protein 5 (*RNF5*) and Flotillin 1 (*FLOT1*)), signaling (*NOTCH4*), RNA metabolism (Coiled-coil alpha-helical rod protein 1 (*CCHCR1*)), lncRNA (*LINC00243* (NONHSAG043434.2), and pseudogenes (WAS protein family member 5, pseudogene (*WASF5P*), Serine/threonine kinase 19B, pseudogene (*STK19B*), Cytochrome P450 family 21 subfamily A member 1, pseudogene (*CYP21A1P*), and Tenascin XA, pseudogene (*TNXA*)).

To identify the tissue-specific expression patterns of the genes associated with rs886424 and rs3129716, we analyzed their relative expression across 53 tissues, using GTEx RNA-seq datasets (Fig 2B). Additionally, we also analyzed the directionality of the eQTL association using average slope values from the GTEx eQTL analysis (Fig 2C). After the hierarchical clustering of the median gene expression values, we found four tissue-specific gene expression clusters. In cluster 1, *CCHCR1* and *PSORS1C1* show high expression in testis (Fig 2B). In this cluster, rs886424 is correlated with increased expression of *CCHCR1* in 9 tissues and decreased expression of *PSORS1C1* in 3 tissues (Fig 2C and 2D). Cluster 2 harbors the 3 MHC class I HLA genes (*HLA-A*, *HLA-C*, and *HLA-G*) as well as *FLOT1*, *PPP1R18*, and the lncRNA *LINC00243* and shows high expression in whole blood, spleen, and lung (Fig 2B). In this cluster, rs886424 is correlated with increased expression of *FLOT1* in 3 tissues, *HLA-C* in 2 tissues, and *LINC00243* in whole blood (Fig 2C and 2D).

rs3129716 is associated with the 6 MHC class II HLA genes (*HLA-DRB1*, *HLA-DRB5*, *HLA-DQB1*, *HLA-DQB2*, *HLA-DMA*, and *HLA-DMB*) in Cluster 3 and 6 genes (2 CYP genes (*CYP21A2* and *CYP21A1P*), 2 pseudogenes (*TNXA* and *STK19B*), and 2 complement factors (*C4A* and *C4B*)) in Cluster 4. Cluster 3 genes show high expression in EBV-transformed lymphocytes, lung, and spleen (Fig 2B). In this cluster, rs3129716 is correlated with increased expression of *HLA-DMA*, *HLA-DQB2*, and *HLA-DMB* in 8 tissues, 7 tissues, and 1 tissue, respectively, and is correlated with decreased expression of *HLA-DQB1*, *HLA-DRB1*, and *HLA-DRB5* in 9 tissues, 1 tissue, and 1 tissue, respectively (Fig 2C and 2D). Genes in cluster 4 show high expression in adrenal gland. *C4A* and *C4B* are also highly expressed in the liver. In this cluster, rs3129716 is correlated with both increased expression of *CYP21A2* and *C4B* in 7 tissues and 4 tissues, respectively, and decreased expression of *C4A*, *CYP21A1P*, *STK19P*, and *TNXA* across 29 tissues, 26 tissues, 1 tissue, and 1 tissue, respectively.

In the pancreas, rs886424 is associated with increased expression of *WASF5P*, and rs3129716 is associated with increased expression of *HLA-DMA* and decreased expression of *C4A* and *HLA-DQB1*. In addition, we found that the largest number of genes with complex and diverse eQTL associations (associations in ≥ 10 tissues) are located in the chromosome 6 HLA region (16 genes, S3 Fig).

Similar to the HLA locus, we found complex transcriptional regulatory circuits at non-HLA loci associated with T1D. For example, at the chromosome 16p11.2 locus harboring 4 high-probability causal enhancer SNP eQTLs (rs4788084, rs62031562, rs743590, and rs762633) and 19 associated genes (Table 1 and Fig 3A). These include an immunity cytokine Interleukin 27 (*IL27*), an immune cytokine receptor SH2B adaptor protein 1 (*SH2B1*), an immunity transcription factor (Nuclear protein 1, transcriptional regulator (*NUPR1*)), two sulfotransferases (Sulfotransferase family 1A member 1 (*SULT1A1*) and *SULT1A2*), two translation factors (Tu translation elongation factor, mitochondrial (*TUFM*), Eukaryotic translation initiation factor 3 subunit C like (*EIF3C*)), two nuclear pore complexes (Nuclear pore complex interacting protein family member B6 (*NPIPB6*) and *NPIPB9*), a transporter Sphingolipid transporter 1 (*SPNS1*), four pseudogenes (Nuclear pore complex interacting protein family member B7 (*NPIPB7*), Cell division cycle 37 pseudogene 1 (*CDC37P1*), *AC138894*.*3*, and *AC145285*.*5*), a non-coding RNA (ATP2A1 antisense RNA 1 (*ATP2A1-AS1*)) and an unknown gene *AC145285*.*2* (Fig 3B). Interestingly, we found that the eQTL rs4788084 lies in a canonical TFBS motif for EBF Transcription Factor 1 (EBF1), indicating that this genetic variant might perturb the binding of EBF1 (Fig 3A and Table 1). Three of the eQTLs, rs762633, rs743590, and rs62031562, are associated with decreased expression of the histone deacetylase complex component SAGA complex associated factor 29 (*SGF29*) in tibial artery, and with increased expression of a protein involved in membrane trafficking, Rabaptin, RAB GTPase binding effector protein 2 (*RABEP2*), in adrenal gland. In contrast, expression of SH3 domain binding kinase 1 (*SBK1*) is associated only with rs4788084.

Although the 19 candidate T1D risk genes in the 16p11.2 locus are expressed at low levels in the pancreas (Fig 3B), the 4 high-probability causal enhancer SNP eQTLs (rs4788084, rs62031562, rs743590, and rs762633) are correlated with increased expression of three genes, *SULT1A2*, *TUFM*, and *CDC37P1*, in 34 tissues, 19 tissues, and 19 tissues, respectively, including the pancreas (Fig 3C). The tissue-specific expression pattern of the 19 genes associated with the 4 eQTLs in the 16p11.2 locus shows two major groups, *SULT1A2*, *SULT1A1*, and *IL27*, which are highly expressed in the liver and *NPIPB6*, *NPIPB7*, *NPIPB9*, *ATP2A1-AS1*, *AC145285*.*5*, and *AC138894*.*3*, which are highly expressed in testis (Fig 3B). Among the cluster 1 genes, we found that the minor alleles of the 4 eQTLs are associated with increased expression of *SULT1A2* and *IL27* across 34 tissues and 2 tissues, respectively, and with decreased expression of *SULT1A1* across 12 tissues (Fig 3C and 3D). In cluster 2, the 4 eQTLs are commonly correlated with increased expression of *AC145285*.*5* across 5 tissues, and decreased expression of *NPIPB7*, *AC138894*.*3*, *NPIPB9* and *ATP2A1-AS1* across 16 tissues, 4 tissues, 2 tissues, and 1 tissue, respectively. In addition, *NPIPB6* shows opposite directional associations with the four eQTLs, in a tissue-specific manner: increased expression in skin sun exposed lower leg and decreased expression in testis. Of the remaining eQTL genes, *TUFM* and *EIF3C* are highly expressed in EBV-transformed lymphocytes and transformed fibroblasts and are associated with increased expression in 19 tissues and 2 tissues, respectively. Taken together, our results indicated that T1D-associated high-probability causal enhancer SNPs mediate genetic risk for the disease through modulation of complex transcriptional regulatory circuits in both HLA and non-HLA T1D risk loci.

## Discussion

In this study, we compiled 1,817 candidate T1D risk SNPs and prioritized 26 as high-probability causal enhancer SNPs by using a comprehensive integrative functional annotation analysis (Fig 1 and Table 1). From the 1,817 candidate T1D SNPs, we found 745 eQTLs and 159 associated genes, with significant enrichments in immune response functions and lymphocyte-specific expression signatures (Table 2, S7 Table and S1 Fig). Of the 26 high-probability causal enhancer SNPs, 15 are eQTLs for 64 associated genes and 4 SNPs reside in lncRNA regions (Table 1). From these 15 high-probability causal enhancer eQTLs, we discovered two complex regulatory circuits at the HLA locus (Fig 2A) and the non-HLA 16p11.2 locus (Fig 3A).

Previously, Nyaga *et al*., [9] investigated 180 GWAS-reported T1D SNPs (*p* ≤ 9E-6) and nominated 246 spatially-regulated genes. Compared with their analysis, the current study has three major differences; 1) although we selected fewer SNPs for analysis, due to employing a more stringent criteria (*p* < 5E-8), our analysis included high-confidence loci, including 33 T1D SNPs identified in the ImmunoChip T1D fine-mapping study [31], as well as a SNP (rs115829748) found from the meta-analysis of three different cohorts [50]; 2) for this integrated analysis we included not only GWAS tag SNPs, but also SNPs in high LD, giving us greater power to discover functional causal variants; 3) we selected high-probability SNPs by incorporating annotations of both known enhancer and TFBS regions. Although 93 T1D SNPs (73% of the 127 GWAS SNPs) are shared between the two studies, these differences lead to largely different results, such as the fact that out of the 26 high-probability causal enhancer SNPs, 22 (77%) were originally selected due to LD relationships, and are unique to our study. Consequently, there is minimal overlap between the sets of nominated T1D risk genes between our studies (S3 and S5 Tables).

The HLA locus has long been known as a major genetic risk region for T1D, as well as an autoimmune disease susceptibility region [51, 52]. In addition, many non-HLA loci also contribute to T1D genetic risk. For example, the variant rs4788084 in the 16p11.2 non-HLA region has been previously reported to be associated with T1D risk, implicating the nearby genes *IL27*, *SULT1A2*, *SH2B1*, *SPNS1*, and *TUFM* [13, 52]. Other studies have highlighted genetic evidence for the importance of genes active in antigen presenting cells (APCs), including at least 17 T1D-associated genes [53]. Among them, three HLA genes, *HLA-A*, *HLA-DQB1*, and *HLA-DRB1*, as well as three non-HLA genes, Cytotoxic T-lymphocyte associated protein 4 (*CTLA4*), *IL27*, and *SH2B1*, were also identified in our analyses. In the 16p11.2 locus, 4 eQTLs that we identified as high-probability causal enhancer SNPs (rs4788084, rs762633, rs743590, and rs62031562) are associated with increased expression of *IL27* (Fig 3). Interestingly, APC-secreted IL27 can modulate the differentiation and activity of various T cell subsets [54]. Consistent with our analysis, these 4 high-probability causal enhancer SNPs were also found to be associated with increased expression of the mitochondrial translation factor *TUFM* in eQTL data from both CD4+ and CD8+ T cells [55]. Intriguingly, the CD4+ T cell eQTL data also supports another of our high-probability causal enhancer SNPs, rs478222 in the 2p23.3 locus, which is associated with decreased expression of a key signaling gene, Adenylate Cyclase 3 (*ADCY3*) in whole blood [55], an association that is further supported by three-dimensional genome organization data [9] (see “Nyaga_2018” and “CD4_cis_eQTLs” columns in S3 and S5 Tables). ADCY3 is a membrane-bound enzyme that hydrolyzes ATP to cyclic AMP (cAMP), and the canonical second messenger cAMP is well known as an inhibitor of T cell activation [56, 57]. This gene has been reported in previous studies [13, 58], but the potential mechanistic relevance for T1D pathogenesis was not considered. In addition, we identified three TCR signaling genes, Linker for activation of T cells (*LAT*), Phosphatase and tensin homolog (*PTEN*) [59], and Protein kinase D2 (*PRKD2*), all of which are associated with increased expression by 1, 7, and 5 candidate T1D eQTLs, respectively. Taken all together, a model emerges whereby these candidate causal enhancer T1D genetic variants might act to increase the propensity of APCs to present autoantigens and secrete IL27, leading to hyperactivation of T lymphocytes by dysregulation of cAMP-mediated inhibition, a situation which could be further exacerbated by increased expression of TCR signaling genes, thus triggering the onset of T1D.

The HLA class I (*HLA-A*, *-B*, and *-C*) and class II (*HLA-DM*, *-DQ*, and *-DR*) genes are expressed on most nucleated cells and APCs, respectively [60]. Hyperexpression of HLA class I in the β-cells of T1D patients has been previously reported [61]. However, we did not find any associations with increased expression of our HLA class I T1D risk genes in the pancreas, where autoantigen presentation by β-cells would be occurring. This result might be explained by the fact that the GTEx RNA-seq data that we used for our analyses did not come from T1D patient samples. In contrast, we did find a significant enrichment of expression of T1D risk locus genes in CD4^+^ and CD8^+^ T cells, as well as CD4^+^CD25^+^ T_REG_ cells and CD19^+^ B cells, as identified by the SNPsea algorithm (S2 Fig). These results support the idea that T1D causal enhancer variants contribute to dysregulation of genes involved in T cell-mediated immune response, playing a causal role in T1D pathogenesis.

Additionally, T1D-associated eQTLs exhibited effects in a broad range of tissues, which may contribute to T1D morbidity and complications. We found T1D-associated eQTLs in the HLA locus have high eQTL effect sizes across ≥ 10 tissues (S3 Fig). Previously, linkage studies have found associations between chronic microvascular complications of T1D and variation in the HLA locus [62, 63]. Although Lipner *et al*. [63] reported the influence of the HLA region to T1D microvascular complications, including retinopathy, nephropathy, and neuropathy from 415 families, genetic risk variants for T1D disease complications still need to be elucidated. Interestingly, a 220 kb (28.74–28.95 Mb range) microdeletion within the 16p11.2 region was reported to be associated with a wide range of disorders, such as developmental delay, autism, epilepsy, congenital anomalies, and obesity [64]. Among the 9 genes (*EIF3C*, *CDC37P1*, *AC145285*.*5*, *NPIPB9*, *AC145285*.*2*, *TUFM*, *SH2B1*, *ATP2A1-AS1*, and *RABEP2*) located in the 16p11.2 region (Fig 3A), *SH2B1* was reported as the causal gene of morbid obesity [65]. Therefore, it is possible that T1D-associated transcriptional regulatory circuits in the 16p11.2 locus contribute to T1D patient morbidity and disease complications.

Multiple clinical strategies have been tested to treat the process of autoreactive T cell-mediated β-cell destruction, including immunotherapies which aimed to deplete endogenous T and B cells, and cell therapies which treated T1D patients with their own T_REG_ cells, which had been expanded ex vivo and re-infused, sometimes in combination with pharmacological agents [66]. Another strategy, hematopoietic stem cell (HSC) therapy, has shown positive results in T1D patients [67]. Interestingly, recent evidence has suggested that deficiencies in programmed death ligand 1 (PD-L1) protein function in HSCs could act as a potential initiator of T1D pathogenesis [68]. Insulin ancillary drugs, which improve glycemic control by targeting sodium glucose co-transporters (SGLTs), have also been tested for T1D treatment [69]. The CTLA4-immunoglobulin fusion protein (Abatacept), which functions by blocking the co-stimulation of T cells, has also yielded positive outcomes as a promising T1D drug [70, 71]. Although the results of our analysis do not implicate the SGLTs or PD-L1, they do support the dysregulated T cell-mediated immune response hypothesis, identifying CTLA4 as an potential therapeutic target, as well as additional targets in T cells (ADCY3, LAT, PTEN, and PRKD2), B cells (C1q and TNF related 6 (C1QTNF6)), and APCs (HLA-A, HLA-DQB1, HLA-DRB1, IL27, and SH2B1).

A limitation of this work is that our integrative analysis was not performed on T1D patient-specific data, which might have led us to miss disease context-specific genetic regulatory effects. Future work in this area will benefit greatly from the T1D patient cell- and tissue-specific genomic and epigenomic resources currently being created and deposited at the previously mentioned T1D Knowledge Portal (https://t1d.hugeamp.org).

In conclusion, in this study, we performed an integrative functional annotation analysis to identify causal enhancer variants associated with T1D genetic risk and the transcriptional regulatory circuits that they affect. Our results indicate that enhancer-based immune dysregulation is likely to contribute to T1D pathogenesis. In addition, complex genetic regulations at both HLA and non-HLA loci potentially contribute to T1D onset and progression. These results suggest that our prioritized risk variants may help to diagnose T1D and can be therapeutic targets for patients.

## Supporting information

S1 Fig Clustering of 159 eQTL-associated T1D risk genes by their tissue-specific expression patterns. (PDF)

S2 Fig Significant enrichment of T1D risk loci gene expression in CD4+ and CD8+ T cells. (PDF)

S3 Fig Expression changes of 35 genes associated with eQTLs affecting over 10 tissues. (PDF)

S1 Table Pairs of GWAS catalog SNPs and LDlink SNPs. (XLSX)

S2 Table Prioritization of 26 highest-probability SNPs. (XLSX)

S3 Table GTEx eQTLs and their associated genes as well as nearest genes associated with T1D SNPs. (XLSX)

S4 Table CoDeS3D results: T1D SNP-gene pairs. (XLSX)

S5 Table 159 eQTL-associated genes and their functional categories. (XLSX)

S6 Table lncRNASNP2: 199 lncRNA-SNP pairs of 78 T1D SNPs and 42 associated lncRNAs. (XLSX)

S7 Table GO analysis of 159 eQTL genes. (XLSX)

S8 Table SNPsea results. (XLSX)
