# Supplementary material for: A comprehensive integrated post-GWAS analysis of Type 1 diabetes reveals enhancer-based immune dysregulation
Source: PLoS One. 2021 Sep 16;16(9):e0257265. doi: 10.1371/journal.pone.0257265 (PMC8445446; doi:10.1371/journal.pone.0257265)
Supplement: S1 Fig — (PDF) [file pone.0257265.s001.pdf]

S1 Figure. Clustering of 159 eQTL-associated T1D risk genes by their tissue-specific expression patterns

To identify tissue-specific gene expression, genes were grouped by the hierarchical clustering algorithm in R (cluster cut distance = 7.6). Out of the 28 clusters, 5 of them are major groups including ≥ 17 genes: group 6 showed brain-specific gene expression; group 7 and 8 displayed EBV-transformed lymphocytes-, lung-, and spleen-specific gene expression. And genes in group 7 also showed high expression in whole blood. Group 11 exhibited testis-specific gene expression. In group 10, 5 genes (ORMDL3, ALDH2, IL27, MST1, and APOM) were highly expressed in the liver. In group 14, 6 genes (GPSM1, VARS2, RASGRP1, AC116158.1, SBK1, and WASF5P) were highly expressed in brain cerebellum (Cerebellum and Cerebellar Hemisphere). In addition, 6 genes (CYP21A2, TNXA, CYP21A1P, STK19B, C4A, and C4B) in group 27 were highly expressed in adrenal gland-specific manner. And 38 genes in cluster 1 and 3 showed pan-organ expressions except for brain, heart, kidney, liver and whole blood with low-levels of gene expression.

Abbreviations: Adipose_1, Adipose - Subcutaneous; Adipose_2, Adipose - Visceral (Omentum); Artery_1, Artery - Aorta; Artery_2, Artery - Coronary; Artery_3, Artery - Tibial; Brain_1, Brain - Amygdala; Brain_2, Brain - Anterior cingulate cortex (BA24); Brain_3, Brain - Caudate (basal ganglia); Brain_4, Brain - Cerebellar Hemisphere; Brain_5, Brain - Cerebellum; Brain_6, Brain - Cortex; Brain_7, Brain - Frontal Cortex (BA9); Brain_8, Brain - Hippocampus; Brain_9, Brain - Hypothalamus; Brain_10, Brain - Nucleus accumbens (basal ganglia); Brain_11, Brain - Putamen (basal ganglia); Brain_12, Brain - Spinal cord (cervical c-1); Brain_13, Brain - Substantia nigra; Cells_1, Cells - EBV-transformed lymphocytes; Cells_2, Cells - Transformed fibroblasts; Cervix_1, Cervix - Ectocervix; Cervix_2, Cervix - Endocervix; Colon_1, Colon - Sigmoid; Colon_2, Colon - Transverse; Esophagus_1, Esophagus - Gastroesophageal Junction; Esophagus_2, Esophagus - Mucosa; Esophagus_3, Esophagus - Muscularis; Fallopian_Tube, Fallopian Tube; Heart_1, Heart - Atrial Appendage; Heart_2, Heart - Left Ventricle; Skin_1, Skin - Not Sun Exposed (Suprapubic); Skin_2, Skin - Sun Exposed (Lower leg)

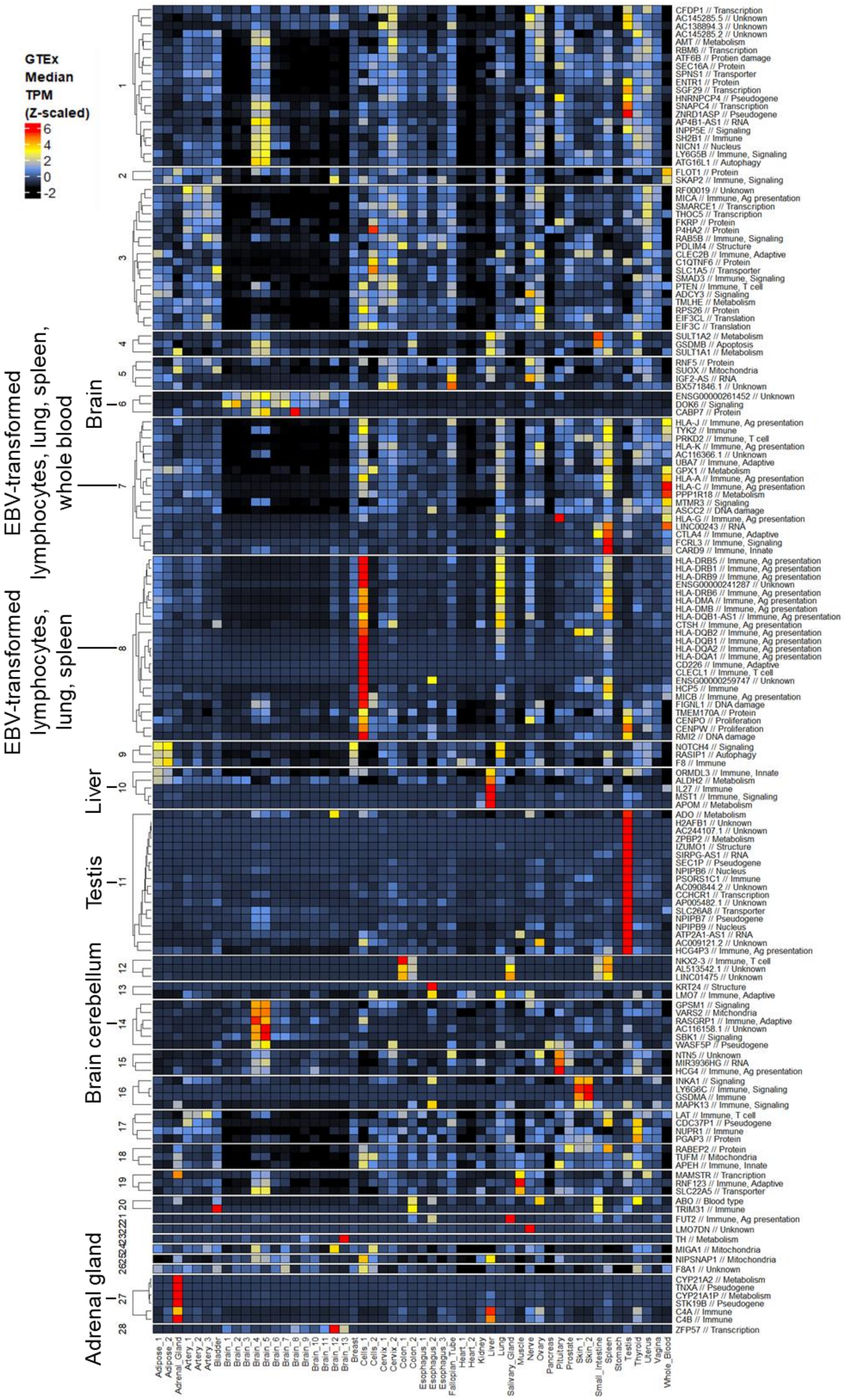
GTEx
Median
TPM
(Z-scaled)
6
4
2
0
-2
Brain
EBV-transformed lymphocytes, lung, spleen, whole blood
EBV-transformed lymphocytes, lung, spleen
Liver
Testis
Brain cerebellum
Adrenal gland
1
2
3
4
5
6
7
8
9
10
11
12
13
14
15
16
17
18
19
20
21
22
23
24
25
26
27
28
CFDP1 // Transcription
AC145285.5 // Unknown
AC138894.3 // Unknown
AC145285.2 // Unknown
AMT // Metabolism
RBM6 // Transcription
ATF6B // Protien damage
SEC16A // Protein
SPNS1 // Transporter
ENTR1 // Protein
SGF29 // Transcription
HNRNPCP4 // Pseudogene
SNAPC4 // Transcription
ZNRD1ASP // Pseudogene
AP4B1-AS1 // RNA
INPP5E // Signaling
SH2B1 // Immune
NICN1 // Nucleus
LY6G5B // Immune, Signaling
ATG16L1 // Autophagy
FLOT1 // Protein
SKAP2 // Immune, Signaling
RF00019 // Unknown
MICA // Immune, Ag presentation
SMARCE1 // Transcription
THOC5 // Transcription
FKRP // Protein
P4HA2 // Protein
RAB5B // Immune, Signaling
PDLIM4 // Structure
CLEC2B // Immune, Adaptive
C1QTNF6 // Protein
SLC1A5 // Transporter
SMAD3 // Immune, Signaling
PTEN // Immune, T cell
ADCY3 // Signaling
TMLHE // Metabolism
RPS26 // Protein
EIF3CL // Translation
EIF3C // Translation
SULT1A2 // Metabolism
GSDMB // Apoptosis
SULT1A1 // Metabolism
RNF5 // Protein
SUOX // Mitochondria
IGF2-AS // RNA
BX571846.1 // Unknown
ENSG00000261452 // Unknown
DOK6 // Signaling
CABP7 // Protein
HLA-J // Immune, Ag presentation
TYK2 // Immune
PRKD2 // Immune, T cell
HLA-K // Immune, Ag presentation
AC116366.1 // Unknown
UBA7 // Immune, Adaptive
GPX1 // Metabolism
HLA-A // Immune, Ag presentation
HLA-C // Immune, Ag presentation
PPP1R18 // Metabolism
MTMR3 // Signaling
ASCC2 // DNA damage
HLA-G // Immune, Ag presentation
LINC00243 // RNA
CTLA4 // Immune, Adaptive
FCRL3 // Immune, Signaling
CARD9 // Immune, Innate
HLA-DRB5 // Immune, Ag presentation
HLA-DRB1 // Immune, Ag presentation
HLA-DRB9 // Immune, Ag presentation
ENSG00000241287 // Unknown
HLA-DRB6 // Immune, Ag presentation
HLA-DMA // Immune, Ag presentation
HLA-DMB // Immune, Ag presentation
HLA-DQB1-AS1 // Immune, Ag presentation
CTSH // Immune, Ag presentation
HLA-DQB2 // Immune, Ag presentation
HLA-DQB1 // Immune, Ag presentation
HLA-DQA2 // Immune, Ag presentation
HLA-DQA1 // Immune, Ag presentation
CD226 // Immune, Adaptive
CLECL1 // Immune, T cell
ENSG00000259747 // Unknown
HCP5 // Immune
MICB // Immune, Ag presentation
FIGNL1 // DNA damage
TMEM170A // Protein
CENPO // Proliferation
CENPW // Proliferation
RMI2 // DNA damage
NOTCH4 // Signaling
RASIP1 // Autophagy
F8 // Immune
ORMDL3 // Immune, Innate
ALDH2 // Metabolism
IL27 // Immune
MST1 // Immune, Signaling
APOM // Metabolism
ADO // Metabolism
H2AFB1 // Unknown
AC244107.1 // Unknown
ZPBP2 // Metabolism
IZUMO1 // Structure
SIRPG-AS1 // RNA
SEC1P // Pseudogene
NPIPB6 // Nucleus
PSORS1C1 // Immune
AC090844.2 // Unknown
CCHCR1 // Transcription
AP005482.1 // Unknown
SLC26A8 // Transporter
NPIPB7 // Pseudogene
NPIPB9 // Nucleus
ATP2A1-AS1 // RNA
AC009121.2 // Unknown
HCG4P3 // Immune, Ag presentation
NKX2-3 // Immune, T cell
AL513542.1 // Unknown
LINC01475 // Unknown
KRT24 // Structure
LMO7 // Immune, Adaptive
GPSM1 // Signaling
VARS2 // Mitochondria
RASGRP1 // Immune, Adaptive
AC116158.1 // Unknown
SBK1 // Signaling
WASF5P // Pseudogene
NTN5 // Unknown
MIR3936HG // RNA
HCG4 // Immune, Ag presentation
INKA1 // Signaling
LY6G6C // Immune, Signaling
GSDMA // Immune
MAPK13 // Immune, Signaling
LAT // Immune, T cell
CDC37P1 // Pseudogene
NUPR1 // Immune
PGAP3 // Protein
RABEP2 // Protein
TUFM // Mitochondria
APEH // Immune, Innate
MAMSTR // Trancription
RNF123 // Immune, Adaptive
SLC22A5 // Transporter
ABO // Blood type
TRIM31 // Immune
FUT2 // Immune, Ag presentation
LMO7DN // Unknown
TH // Metabolism
MIGA1 // Mitochondria
NIPSNAP1 // Mitochondria
F8A1 // Unknown
CYP21A2 // Metabolism
TNXA // Pseudogene
CYP21A1P // Metabolism
STK19B // Pseudogene
C4A // Immune
C4B // Immune
ZFP57 // Transcription
Adipose_1
Adipose_2
Adrenal_Gland
Artery_1
Artery_2
Artery_3
Bladder
Brain_1
Brain_2
Brain_3
Brain_4
Brain_5
Brain_6
Brain_7
Brain_8
Brain_9
Brain_10
Brain_11
Brain_12
Brain_13
Breast
Cells_1
Cells_2
Cervix_1
Cervix_2
Colon_1
Colon_2
Esophagus_1
Esophagus_2
Esophagus_3
Fallopian_Tube
Heart_1
Heart_2
Kidney
Liver
Lung
Salivary_Gland
Muscle
Nerve
Ovary
Pancreas
Pituitary
Prostate
Skin_1
Skin_2
Small_Intestine
Spleen
Stomach
Testis
Thyroid
Uterus
Vagina
Whole_Blood
