# Supplementary material for: A comprehensive integrated post-GWAS analysis of Type 1 diabetes reveals enhancer-based immune dysregulation
Source: PLoS One. 2021 Sep 16;16(9):e0257265. doi: 10.1371/journal.pone.0257265 (PMC8445446; doi:10.1371/journal.pone.0257265)

S2 Figure. Significant enrichment of T1D risk loci gene expression in CD4+ and CD8+ T cells

To identify cell type enrichment of expression of genes in T1D risk loci, the SNPsea algorithm was applied to the 1,817 candidate T1D SNPs. Empirical *P* values of the top 25 tissues and Pearson correlation coefficients for pairs of cell types were displayed. T1D risk loci genes were significantly enriched in both CD4+ T cells and CD8+ T cells in both the FANTOM5 ($P < 0.0001$, highlighted in yellow) (**A**) and GeneAtlas2004 datasets ($P < 0.0005$, highlighted in yellow) (**B**). In addition, enrichment in CD4+CD25+ regulatory T cells in the FANTOM5 dataset and in B cells in the GeneAtlas2004 dataset were also significant (highlighted in yellow).

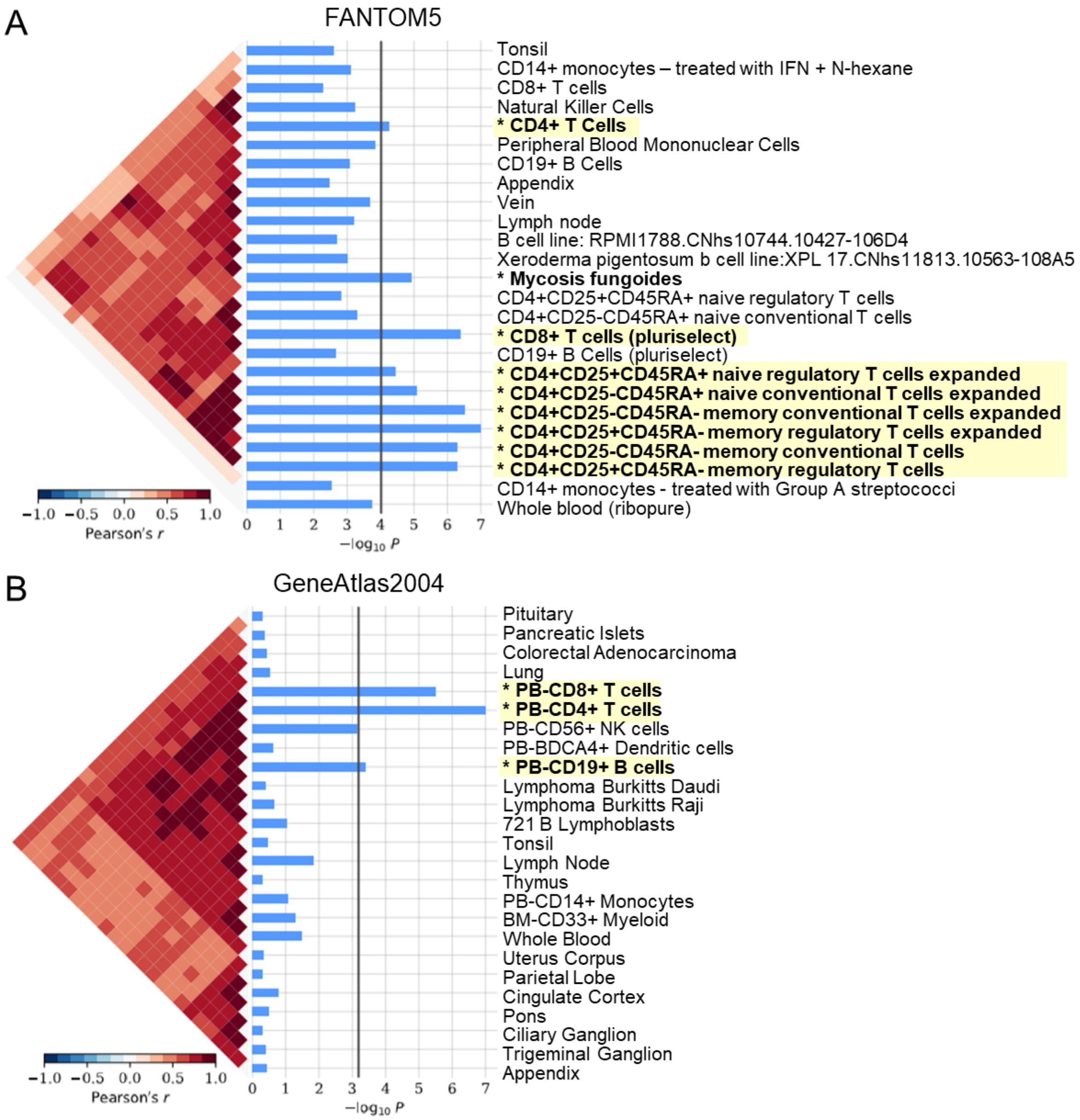

Supplement: S2 Fig — (PDF) [file pone.0257265.s002.pdf]
