# Supplementary material for: A comprehensive integrated post-GWAS analysis of Type 1 diabetes reveals enhancer-based immune dysregulation
Source: PLoS One. 2021 Sep 16;16(9):e0257265. doi: 10.1371/journal.pone.0257265 (PMC8445446; doi:10.1371/journal.pone.0257265)

S3 Figure. Expression changes of 35 genes associated with eQTLs affecting over 10 tissues

Direction-specific effect sizes of T1D-associated eQTLs that have associations in ≥ 10 tissues, displayed as a heatmap using average slope values of the eQTL-gene pair association in each tissue. The tissues highlighted in yellow show high expression of HLA genes (see **S1 Figure**, group 7 and 8). Pancreas is highlighted in green.

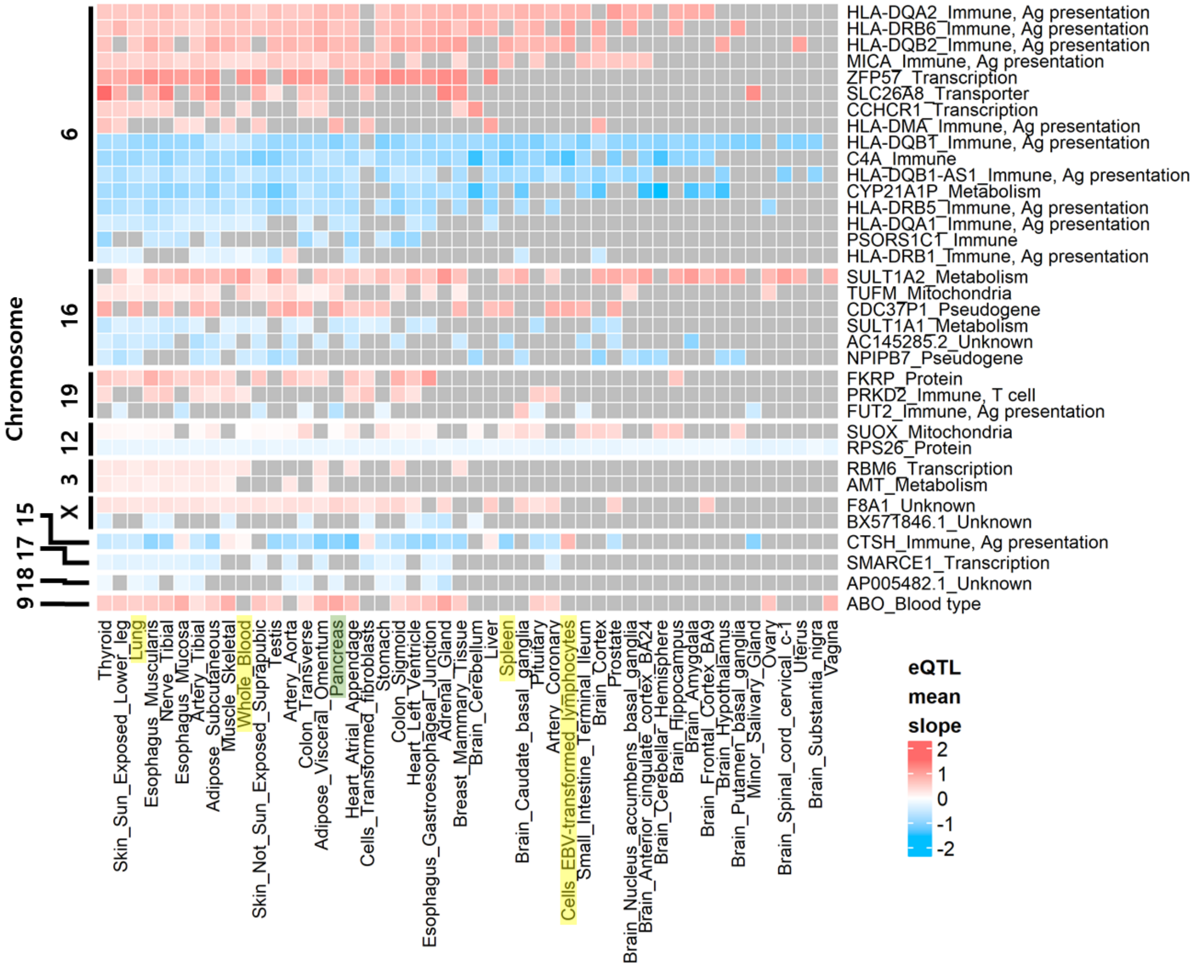

Supplement: S3 Fig — (PDF) [file pone.0257265.s003.pdf]
